# Fcα Receptor Type I and Its Association with Atherosclerosis Development

**DOI:** 10.14789/jmj.JMJ23-0003-OA

**Published:** 2023-05-20

**Authors:** YUYA DESAKI, YUTAKA KANAMARU, RENATO MONTEIRO, YUSUKE SUZUKI

**Affiliations:** 1 Department of Nephrology, Juntendo University Faculty of Medicine, Tokyo, Japan; 1 Department of Nephrology, Juntendo University Faculty of Medicine, Tokyo, Japan; 2 Center for Research on Inflammation, Université de Paris, Paris, France; 2 Center for Research on Inflammation, Université de Paris, Paris, France

**Keywords:** atherosclerosis, oxidized low-density lipoprotein, mitogen-activated protein kinase signaling, macrophage foam cell, FcαRI

## Abstract

**Objectives:**

Atherosclerosis is a chronic inflammatory disease characterized by lipid accumulation and local inflammation, which are regulated by the immune system. The immunological aspects of this disease are unclear. Immunoglobulin A regulates many cell responses through interactions with Fcα receptor type I (FcαRI). Anti-FcαRI antibody inhibits activating receptors by inducing an inhibitory immunoreceptor tyrosine-based activation motif configuration. However, the role of FcαRI in atherosclerosis development is unclear. Here, we investigated the utility of FcαRI targeting to induce inhibitory immunoreceptor tyrosine-based activation motif signaling in atherosclerosis treatment.

**Materials:**

ApoE^-/-^ transgenic mice expressing the FcαRIR209L/FcRγ chimeric protein (FcαRIR209L/FcRγApoE^-/-^ mice) were generated. We prepared an FcαRIR209L/FcRγ transfectant (I3D) from a mouse macrophage cell line (RAW264.7).

**Methods:**

Anti-FcαRI or control antibody was used to investigate a high-fat-diet-induced FcαRIR209L/FcRγApoE^-/-^ mouse model of atherosclerosis. The antibody was also used to assess macrophage foam cell formation via Oil Red O staining and mitogen-activated protein kinase signaling via immunoblotting in the FcαRIR209L/FcRγ-expressing RAW264.7 macrophage cell line I3D.

**Results:**

Targeting of monovalent FcαRI induced inhibitory effects in the FcαRIR209L/FcRγApoE^-/-^ mouse model of atherosclerosis by inhibiting macrophage infiltration. FcαRI targeting using the anti-FcαRI antibody also reduced mitogen-activated protein kinase signaling and foam cell formation, leading to decreased interleukin (IL)-1b and monocyte chemoattractant protein (MCP)-1.

**Conclusions:**

We demonstrated that targeting monovalent FcαRI suppresses atherosclerosis development. These findings can support the future clinical exploration of FcαRI targeting for atherosclerosis treatment.

## Introduction

Atherosclerosis is a chronic disease with a multifactorial etiology that ultimately leads to the development of rupture-prone plaques and atherothrombotic events^1)^. Clinical trials and animal experiments have supported the notion that advanced plaques share common properties including augmented lipid- rich necrotic core and macrophage accumulation^2)^. Macrophages often accumulate in various regions of vulnerable plaques and are the main source of cytokines^3)^. During the last decade, oxidized low- density lipoprotein (ox-LDL) and its interactions with monocytes/macrophages were considered the primary atherogenic components in dyslipidemia^4)^. The concentration of ox-LDL is markedly elevated in atherosclerotic lesions and reaches cytotoxic levels and subsequent inflammatory events^3)^. Mitogen-activated protein kinase (MAPK)-induced phosphorylation events play important roles in macrophage migration in plaques^5)^. Intracellular MAPK signaling cascades are involved in the pathogenesis of cardiac and vascular diseases. In macrophages, an interaction between CD36 and ox-LDL induces the phosphorylation of Lyn, one of several Src-family tyrosine kinases in immune cells, and subsequent activation of extracellular signal-regulated kinase (ERK) and p38 mediates the uptake of ox-LDL^6)^, leading to inhibition of MAPK signaling and attenuation of foam cell formation^7)^. Ox-LDL induces vascular smooth muscle cell proliferation and inflammation with the foam cell formation^8)^. Since MAPK signaling pathway affects foam cell aggregation and inflammatory responses, inhibition of nuclear factor-κB and MAPK signaling attenuates atherosclerosis^9)^.

Fc*α* receptor type I (Fc*α*RI; CD89) is the only Fc receptor specific to immunoglobulin A (IgA) expressed on myeloid cells, including macrophages, monocytes, dendritic cells, Kupffer cells, neutrophils, and eosinophils^10)^. Fc*α*RI is expressed in the presence or absence of a physical association with the Fc*γ*R adaptor, which contains an immunoreceptor tyrosine-based activation motif (ITAM). Fc*α*RI, a unique member of the FcR family, exerts a dual role in immune cell inhibition and activation^10, 11)^. It is known that an inhibitory signal is generated when monomeric IgA (mIgA) binds to two Fc*α*RI, while an active signal is required the binding of IgA-immune complexes to Fc*α*RI. Serum IgA is generated mainly as a monomeric form (about 85% to 90% of total serum IgA) by bone marrow plasma cells. Therefore, we think that an inhibitory signal of Fc*α*RI is dominant at least in circulation. An inhibitory ITAM (ITAMi) configuration induced by monovalent targeting of Fc*α*RI (anti-Fc*α*RI Fab fragment) initiates the recruitment of Src homology domain 2-containing protein- tyrosine phosphatase-1 (SHP-1), which has inhibitory potential^12)^. This step leads to the deactivation of the inflammatory reaction, thereby preventing autoimmune processes. Previous studies demonstrated the involvement of Fc*γ*R signal activation through the ITAM-containing Fc*γ*R adaptor in diseases^5, 13)^. The anti-Fc*α*RI fragment antigen- binding (Fab) region negatively regulates the magnitude of the innate immune response and has been used as an anti-inflammatory drug to treat kidney diseases^14)^. Furthermore, the inhibitory signal induced by anti-Fc*α*RI Fab in the Fc*α*RIR209L/FcR*γ* chimeric receptor is more potent than that in wild-type Fc*α*RI, which is expressed in the presence or absence of a physical association with the Fc*γ*R adaptor.

Fc*α*RI targeting can halt disease progression and lupus activation by selectively inhibiting cytokine production, leukocyte recruitment, and renal inflammation^15)^. Fc*α*RI-mediated inhibition can suppress several inflammatory diseases in mice, including asthma and glomerulonephritis. Intravenous mIgA and anti-Fc*α*R monovalent antibodies are promising tools for immunotherapy^16)^. In this study, we aimed to evaluate whether Fc*α*RI targeting can prevent atherosclerosis.

## Materials and Methods

### Animals

The mice were bred and maintained in the mouse facilities of the Research Institute for Diseases of Old Age (Juntendo University School of Medicine, Tokyo, Japan). All experiments were conducted in accordance with national guidelines and were approved by a local ethics committee(Juntendo University School of Medicine Animal Experiment Committee; the approval number is 270258).

### Production of the construct, generation of Fc*α*RIR209L/FcR*γ* transgenic (Tg) ApoE^−/−^ mice, and preparation of Fc*α*RIR209L/FcR*γ* transfectant

**Figure 1:**
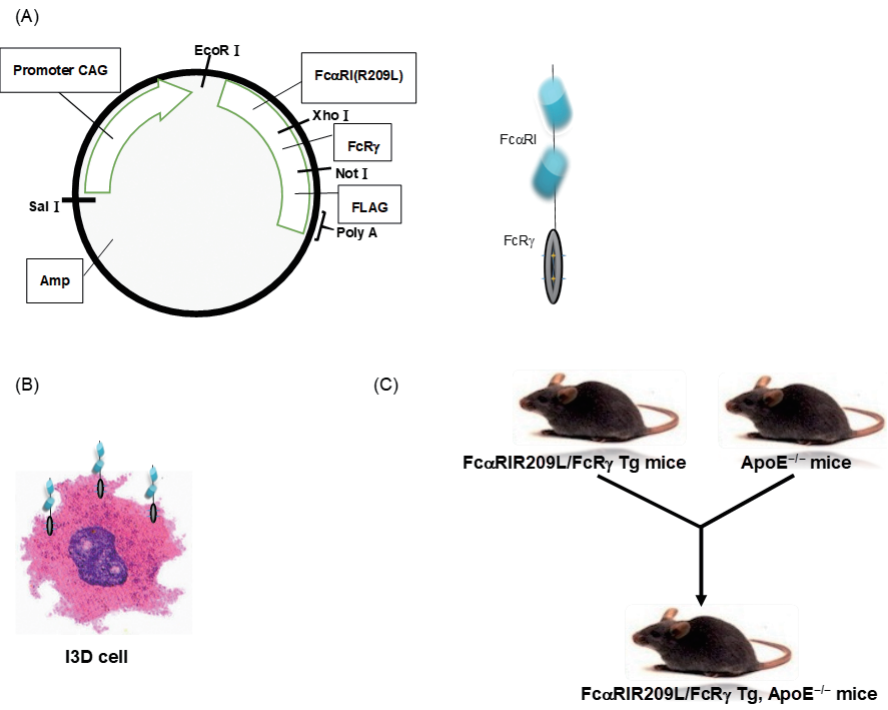
Generation of a mouse macrophage transfectant expressing high levels of FcαRIR209L/FcRγ (I3D cells), FcαRIR209L/FcRγ transgenic (Tg) mice, and FcαRIR209L/FcRγ Tg ApoE^−/−^ mice The transgene consists of cDNA encoding human FcαRIR209L/FcRγ-FLAG and construct containing the mouse β-actin promoter (A). Simplified schematic of FcαRIR209L/FcRα. RAW264.7 macrophages (I3D cells) express high levels of human-28 CD89 on the cell surface (B). Tg mice expressing FcαRI/FcRγ were bred on a C57BL/6J background and then crossed with ApoE^−/−^ mice bred on a C57BL/6J background (C).

A construct encoding human Fc*α*RIR209L/FcR*γ*- FLAG was obtained by inserting an 1165-bp cDNA fragment into the EcoRI site of a CAG promoter containing *β*-actin (UniTeck, Kashiwa, Japan). The transgenic mouse contained human Fc*α*RIR209L/FcR*γ*-FLAG cDNA obtained via the polymerase chain reaction of tail DNA using the transgene-specific primers 5′-GGGTCATTAGTTCATAGCC-3′ and 5′-GGCATATGATACACTTGAT-3′. To determine whether inhibitory Fc*α*RI diminishes the progression of atherosclerosis, Fc*α*RIR209L/FcR*γ* transgenic (Tg) ApoE^−/−^ mice were generated. All mice used in this study were bred and housed under strictly controlled specific pathogen-free conditions. We prepared Fc*α*RIR209L/FcR*γ* transfectant (I3D) cells from a mouse macrophage cell line (RAW264.7) using a Cell Line Optimization Nucleofector Kit (Lonza, Basel, Switzerland) (Figure 1).

### Cell culture

RAW264.7 macrophages were cultured in Glutamax (Invitrogen, Carlsbad, CA, USA) supplemented with 10% fetal calf serum, 100 U/mL penicillin, and 100 mg/mL streptomycin at 37°C with 5% CO_2_ in a humidified incubator. Stable transfectants were selected by adding geneticin (1 mg/m; Sigma Aldrich, St. Louis, MO, USA).

### Animal study protocol

Ten male Fc*α*RIR209L/FcR*γ* transgenic (Tg) ApoE^−/−^ mice (12-week-old) with a C57BL/6J background were used. The animals were fed a diet containing 15% cocoa butter and 0.25% cholesterol, which was obtained from the Animal Center of Juntendo University. After anesthesia (40 mg/kg pentobarbital sodium intraperitoneally), a constrictive silastic tube (0.30 mm), inserted via the caudal vein, was used to elicit plaque formation^17)^. The mice were divided into two groups (n = 5 per group): group 1, Fc*α*RIR209L/FcR*γ* Tg ApoE^−/−^ mice were administered 20 µg of control Fab in 200 µL of saline once daily via the caudal vein for three months; group 2, Fc*α*RIR209L/FcR*γ* Tg ApoE^−/−^ mice were administered 20 µg of A77 (anti-Fc*α*RI antibody) Fab in 200 µL of saline once daily via the caudal vein for three months. Serum samples and aorta tissues were collected at the end of the study.

### IgGs and antibodies

A BALB/c-derived (IgG1) mouse monoclonal antibody (Ab) specific for Fc*α*RI (clones A77 or A59)^18)^ was used as the Fab fragment. Mouse IgG (Jackson Laboratories, Bar Harbor, ME, USA); rabbit anti-phospho ERK MAPK; p38 and c-Jun N-terminal kinase (JNK) antibodies (Cell Signaling Technology, Danvers, MA, USA); and rat anti-mouse F4/80Ab (AbD Serotec, Oxford, UK) were used.

### Foam cell formation and Oil Red O staining

I3D cells (1 × 10^6^ cells/well) were seeded into 6-well plates and stimulated with ox-LDL (100 µg/mL for 24 h; Yiyuan, Guangzhou, China) in the presence or absence of A77 Fab (100 µg/mL for 12 h). Subsequently, the cells were washed with phosphate-buffered saline (PBS) and stained with Oil Red O (Sigma Aldrich Chemicals). Stained (red) foam cells were imaged under a microscope at 40× magnification.

### Western blot analysis

I3D cells were preincubated with A77, A59, or control (Ctrl) Fab (100 μg/mL) for 12 h. The cells were then stimulated with ox-LDL (100 μg/mL) for 20 min, and phosphorylation of ERK, P38, and JNK was assessed using western blotting. Briefly, cultured cells were washed twice with ice-cold PBS and solubilized by incubation at 4°C for 10 min in lysis buffer (50 mM HEPES [pH 7.4], 0.3% Triton X-100, 50 mM NaF, 50 mM NaCl, 1 mM Na_3_VO_4_, 30 mM Na_4_P_2_O_7_, 50 U/mL aprotinin, and 10 mg/mL leupeptin). The protein concentration of the soluble extracts was determined using a protein assay kit (Bio-Rad, Hercules, CA, USA). The collected samples were mixed with a sample buffer (312.5 mmol/L Tris-HCl [pH 6.8], 10% sodium dodecyl sulfate, 50% glycerol, 10% 2-mercaptoethanol, and 0.025% bromophenol blue), heated at 95°C for 5 min before electrophoresis, resolved via sodium dodecyl sulfate-polyacrylamide gel electrophoresis on a 10% acrylamide gel, and transferred to polyvinylidene difluoride membranes. The blots were analyzed as described previously^10)^.

### Immunohistochemical staining

For light microscopy, the sections of mouse aorta tissues were sectioned at 3 μm, paraffin-embedded, and stained using the periodic acid–Schiff reagent. For immunohistochemical staining, frozen mouse aorta tissues were sectioned at 3 μm, fixed in -20°C acetone, and blocked by incubation in a blocking solution (PBS [pH 7.2] containing 2.0% bovine serum albumin, 2% fetal calf serum, and 0.2% fish gelatin at room temperature) for 60 min. Histological features were graded, and F4/80+ cells were counted blindly. A minimum of 10 equatorially sectioned aortas were assessed per animal. The results are expressed as the number of cells per high-power field, which was quantified using a KS-400 version 4.0 image analysis system (KS-400; Carl Zeiss Vision, Oberkochen, Germany).

### Enzyme-linked immunosorbent assay (ELISA)

Blood samples were collected from each mouse from the retro-orbital venous plexus under general anesthesia by inhalation of ether at the end of the study. Interleukin (IL)-1b and monocyte chemoattractant protein (MCP)-1 levels were measured using commercially available ELISA kits (R&D Systems, Minneapolis, MN, USA) according to the manufacturer's protocol.

### Data presentation

All experiments were repeated more than three times, and representative results are shown. Data are expressed as the mean ± 2 standard error. Statistical analyses were performed using the Student's unpaired *t*-test (specifically, for immunoblotting determination, we compared the results with those of each respective control) and analysis of variance. p < 0.05 was considered to indicate statistical significance.

## Results

### Monovalent targeting of Fc*α*RI decreases ox- LDL-induced foam cell formation in Fc*α*RIR209L/FcR*γ* (I3D) cells

**Figure 2:**
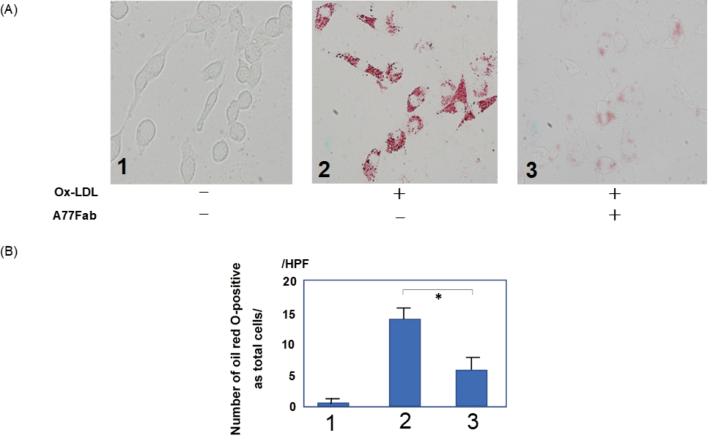
Monovalent targeting of FcαRI decreases foam cell formation in FcαRIR209L/FcRγ (I3D) cells I3D cells were stimulated with oxidized low-density lipoprotein (ox-LDL) in the presence or absence of A77 Fab (A). Oil Red O staining indicated the presence of foam cells. I3D cells were stimulated with ox-LDL (100 μg/mL for 24 h) in the presence or absence of A77 Fab (100 μg/mL for 12 h). Stained (red) foam cells were imaged under a microscope at 40× magnification. In I3D cells stimulated with ox-LDL with A77 Fab, the number of macrophage foam cells was significantly reduced, as demonstrated by Oil Red O staining. The number of Oil Red O-positive cells was expressed as the percentage of total cells (B). Results were obtained from three independent experiments (p < 0.05). HPF, high-power field.

Fc*α*RI-FcR*γ* ITAMi function can be triggered in the absence of co-aggregation. Therefore, we predicted that monovalent targeting, in addition to inhibiting co-expressed ITAM-bearing receptors, affects the responses of receptors involved in different signaling pathways^11)^. We analyzed the effect of anti-Fc*α*RI Fab A77 pretreatment on the foaming response of Fc*α*RIR209L/FcR*γ* (I3D) to ox-LDL. Oil Red O staining showed that A77 Fab, but not the Ctrl Fab, markedly inhibited ox-LDL-induced foam cell formation (Figure 2).

### Ox-LDL-mediated MAPK signaling in Fc*α*RIR209L/FcR*γ* (I3D) cells

**Figure 3:**
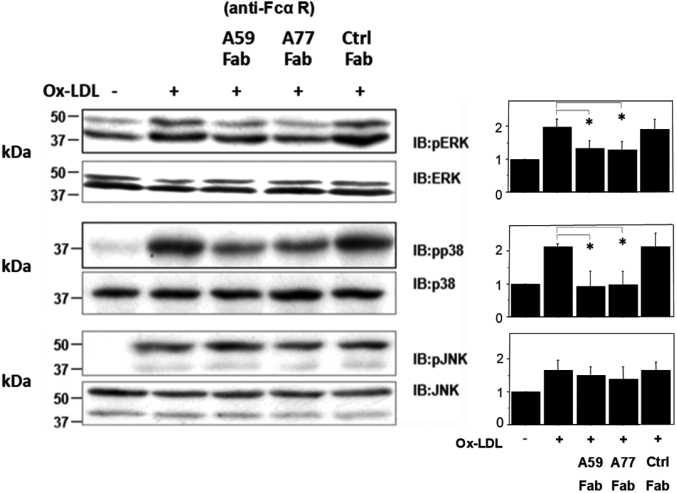
Oxidized low-density lipoprotein (Ox-LDL)-mediated mitogen-activated protein kinase (MAPK) in FcαRIR209L/FcRγ (I3D) cells I3D cells were preincubated with A77 Fab, A77 Fab, or Ctrl Fab (100 μg/mL for 12 h). The cells were then stimulated with ox-LDL (100 μg/mL) for 20 min, and phosphorylation of extracellular signal-regulated kinase (ERK), p38, and c-Jun N-terminal kinase (JNK) was assessed. Western blot analysis showed that ox-LDL-induced MAPK activation was strongly abolished by A77 Fab treatment in I3D cells. Re-probing with JNK, p38, and p42–p44 ERK MAPK is shown as controls for equal loading. Results were obtained from three independent experiments (p < 0.05).

Next, we analyzed the effect of anti-Fc*α*RI (A77 Fab) pretreatment on MAPK in response to ox- LDL in I3D cells. Key events in ox-LDL-mediated signaling, such as JNK, p38, and p42–p44 ERK MAPK phosphorylation, as evaluated by immunoblotting using phospho-specific antibodies, are shown in Figure 2. Phosphorylation was strongly inhibited in I3D cells after preincubation with A77 Fab but not after incubation with Ctrl Fab (Figure 3).

### Oil Red O staining of the aorta of wild-type ApoE-deficient/Fc*α*RIR209L/FcR*γ* phenotype mice fed a high-fat diet for three months

**Figure 4:**
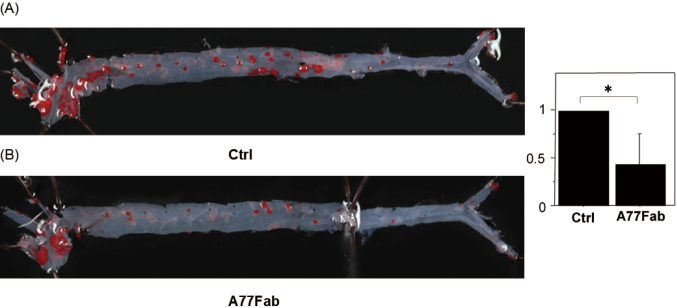
Oil red O staining of the aorta of wild-type ApoE-deficient/FcαRIR209L/FcRγ phenotype mice fed a high-fat diet for three months Wild-type ApoE-deficient/FcαRIR209L/FcRγ mice were generated, fed a high-fat diet for three months, and injected with A77 Fab or PBS Ctrl. Staining the mouse aortas with Oil Red O showed that the expression of A77 Fab (A) was lower than that of PBS Ctrl (B). Results were obtained from three independent experiments (p < 0.05).

Fc*α*RIR209L/FcR*γ* Tg ApoE^−/−^ mice were administered 20 µg of Ctrl Fab in 200 µL of saline once daily via the caudal vein for three months; in group 2, Fc*α*RIR209L/FcR*γ* Tg ApoE^−/−^ mice were administered 20 µg of Ctrl Fab in 200 µL of saline once daily via the caudal vein for three months. Staining the mouse aortas using Oil Red O showed that staining levels were lower in the A77 Fab treatment group than in the Ctrl Fab treatment group (Figure 4).

### Fc*α*RI targeting reduces leukocyte infiltration in A77 Fab-treated mice

**Figure 5:**
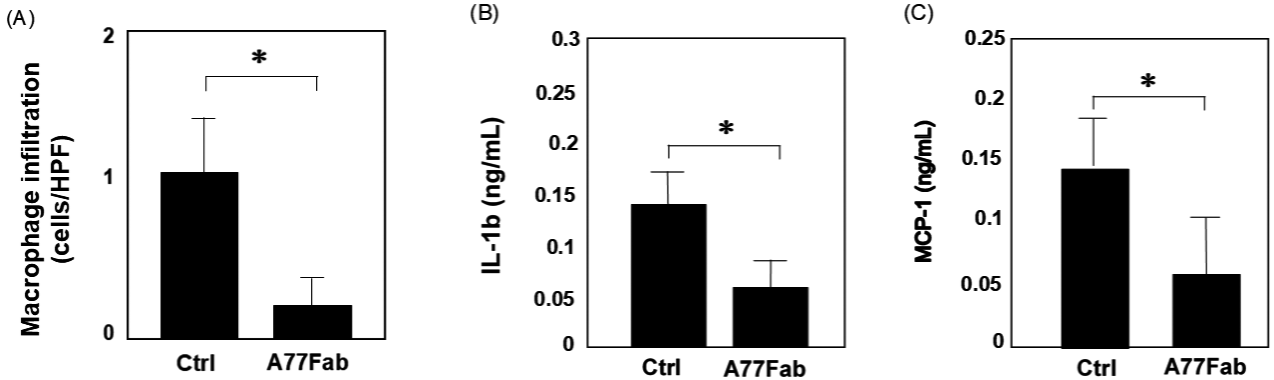
FcαRI targeting reduces leukocyte infiltration and blocks serum cytokine and chemokine production stimulated in an atherosclerosis model Immunohistological analysis of aortic sections from each animal group using anti-mouse F4/80 Ab. The number of infiltrating macrophages is shown (A). Serum interleukin (IL)-1β (B) and monocyte chemoattractant protein-1 (MCP-1) (C) production in each group was measured using an enzyme-linked immunosorbent assay (ELISA). Anti-FcαRI Fab-injected group showed lower protein production compared to the control Fab-injected group (p < 0.05). HPF, high-power field.

To determine whether monovalent targeting of anti-Fc*α*R has therapeutic implications for high-fat diet-induced atherosclerosis, we analyzed the effect of A77 Fab treatment in a high-fat-diet-induced Fc*α*RIR209L/FcR*γ* Tg ApoE^−/−^ mouse model of atherosclerosis. Control antibody-treated animals showed high CD11b+/F4/80+ macrophage infiltration into the aortic tissues (Figure 4). However, A77 Fab-treated mice showed decreased infiltration of aortic tissues by CD11b+/F4/80+ macrophages compared to that in control antibody-treated animals. Thus, A77 Fab treatment showed marked efficacy against atherosclerosis induced by a high-fat diet in Fc*α*RIR209L/FcR*γ* Tg ApoE^−/−^ mice (Figure 5).

### Fc*α*RI monomeric targeting blocks serum cytokine and chemokine production stimulated in atherosclerosis

To examine whether increased aortic macrophage infiltration in Fc*α*RIR209L/FcR*γ* ApoE^−/−^ mice was correlated with serum cytokine and chemokine levels, we performed ELISA using serum isolated from the affected mice. At the end of the study, treatment with the control antibody significantly increased IL-1*β* and MCP-1 secretion. In contrast, the A77 Fab treatment decreased IL-1*β* and MCP-1 levels (Figure 5).

## Discussion

In a previous study, RAW264.7 cells were stimulated with lipopolysaccharide or ox-LDL to mimic the development of atherosclerosis^7)^. To assess the involvement of Fc*α*RI/FcR*γ* in the inhibitory process, we generated a chimeric receptor plasmid by fusing the extracellular and R209L transmembrane domains of Fc*α*RI to the intracytoplasmic tail of human FcR*γ*^14)^. We also generated transfectants expressing Fc*α*RIR209L associated with FcR*γ* (I3D) in RAW 264.7 macrophages^14)^. Several previous studies demonstrated that inhibitory signaling by myeloid Fc*α*RI is a promising anti-inflammatory candidate for treating inflammatory diseases^10)^. However, data supporting its inhibitory effects on atherosclerosis are lacking.

Although the presence of anti-ox-LDL IgG has been well-documented in clinical and animal studies, the role of Fc*γ*Rs in the progression of atherosclerosis remains unclear. The role of activating Fc*γ*R in the progression of atherosclerosis using apoE-Fc*γ*-chain double-knockout mice was examined^19, 20)^. In apoE knockout mice, arterial lesion formation was significantly decreased in apoE-Fc*γ*-chain double-knockout mice.

We also conducted an additional *in vivo* experiment using a Tg mouse with an ApoE-deficient/Fc*α*RIR209L/FcR*γ* phenotype under high-fat diet feeding, which exhibited severe atherosclerotic lesions. The results showed that monovalent targeting of Fc*α*RI in Tg mice with the ApoE-deficient/Fc*α*RIR209L/FcR*γ* phenotype significantly diminished aortic lesions by an inhibitory ITAM (ITAMi) configuration induced using monovalent targeting of Fc*α*RI through the inhibition of macrophage infiltration at the aortic lesion and decreased IL-1*β* and MCP-1 levels.

A previous report assessed atherosclerotic lesions of apoE-inhibitory Fc*γ*RIIb double-knockout mice (apoE-Fc*γ*RIIb (-/-))^21)^, and contrary to their hypothesis, when compared with the apoE single knockout mice, arterial lesions were significantly decreased in apoE-Fc*γ*RII (-/-) mice. Chimeric mice generated by transplanting apoE-Fc*γ*RIIb (-/-) marrow into apoE single knockout mice also developed smaller lesions. Macrophages from Fc*γ*RIIb (-/-) mice produced more IL-1*β* and MCP-1. The mechanisms of this discrepancy remain unknown.

We observed that monovalent targeting of Fc*α*RI was inhibited in an *in vitro* model of ox-LDL- induced foam cell formation. Ox-LDL-induced foam cell formation was markedly decreased in the A77 Fab- or A57 Fab-treated groups compared with that in the Ctrl Fab-treated group. The expression of phosphorylated ERK and p38 was also decreased in the A77 Fab- and A57 Fab-treated groups compared with that in the Ctrl Fab-treated group. Interestingly, phosphorylated JNK expression did not significantly differ between the A77 Fab- or A57 Fab-treated groups and the Ctrl Fab- treated group after stimulation with ox-LDL in Fc*α*RIR209L/FcR*γ* chimeric receptor transfectant macrophages. In line with these findings, inhibitory signaling by myeloid Fc*α*RI significantly decreased the phosphorylation levels of MAPK during atherosclerosis development. The anti-atherogenic properties of inhibitory signaling by myeloid Fc*α*RI observed in I3D cells may be explained by its inhibitory effects on monocyte adhesion, oxidative stress, and the inflammatory response mediated by the ox-LDL/MAPK (ERK1/2/p38) signaling pathway, which was independent of JNK in macrophages.

A previous study demonstrated that IL-1*β* is upstream of the disrupted intestinal barrier function in a mouse model of Kawasaki disease vasculitis, which showed IgA vasculitis development and cardiac inflammation following genetic and pharmacological inhibition of IL-1*β* signaling^22)^. Targeting mucosal barrier dysfunction and the IL-1*β* pathway may also apply to other IgA-related diseases, including IgA vasculitis, IgA nephropathy, and atherosclerosis. Elevated levels of circulating secretory IgA may promote atherosclerosis in Kawasaki disease.

Our study had some limitations. First, our *in vivo* physiological data are not sufficient. Unfortunately, this study did not check serum levels of cholesterol and ox-LDL in the mice. In our future study, we should check them and evaluate this point. However, we carefully reviewed optimal animal models in which the inhibitory effect of myeloid Fc*α*RI in atherosclerosis development has been confirmed. Second, although inhibitory signaling by myeloid Fc*α*RI showed multifunctional potential *in vitro* in both our and previous studies, the contribution of SH2-containing phosphatase SHP-1 recruitment should be evaluated. SHP-1 should be immunoprecipitated to demonstrate this association, and FcR*γ* co-immunoprecipitates in macrophages following treatment with anti-Fc*α*RI Fab should be examined.

We demonstrated that monovalent targeting of Fc*α*RI suppresses atherosclerosis development. These results indicate that the inhibitory signals of Fc*α*RI require FcR*γ* single association. Fc*α*RI is a complex receptor, and the balance in Fc*α*RI targeting is associated with the development of atherosclerosis. These results demonstrate the potential of inhibitory signaling by myeloid Fc*α*RI as a therapeutic approach for atherosclerosis.

## Funding

The authors received no financial support for the research.

## Author contributions

All authors have made substantial contributions to the manuscript. The details are follows: YD: Conceptualization. Methodology. Formal analysis. Investigation. Data curation. Visualization. Project administration. Writing – original draft. Writing – review and editing. YK: Resources. Writing – editing. Supervision. RM: Resources. Writing – editing. Supervision. YS: Conceptualization. Methodology. Data curation. Writing – review and editing. Supervision. All authors approved the final version of the manuscript to be submitted.

## Conflicts of interest statement

The authors declare that there are no conflicts of interest.
